# Chemical Biology Gateways to Mapping Location, Association, and Pathway Responsivity

**DOI:** 10.3389/fchem.2019.00125

**Published:** 2019-03-21

**Authors:** Marcus J. C. Long, Xuyu Liu, Yimon Aye

**Affiliations:** ^1^Independent Researcher, Beverley, United Kingdom; ^2^École Polytechnique Fédérale de Lausanne, Institute of Chemical Sciences and Engineering, Lausanne, Switzerland

**Keywords:** chemical biology methods, T-REX, G-REX, APEX, Bio-ID, PUP-IT, Model organisms

## Abstract

Here we discuss, how by applying chemical concepts to biological problems, methods have been developed to map spatiotemporal regulation of proteins and small-molecule modulation of proteome signaling responses. We outline why chemical-biology platforms are ideal for such purposes. We further discuss strengths and weaknesses of chemical-biology protocols, contrasting them against classical genetic and biochemical approaches. We make these evaluations based on three parameters: occupancy; functional information; and spatial restriction. We demonstrate how the specific choice of chemical reagent and experimental set-up unite to resolve biological problems. Potential improvements/extensions as well as specific controls that in our opinion are often overlooked or employed incorrectly are also considered. Finally, we discuss some of the latest emerging methods to illuminate how chemical-biology innovations provide a gateway toward information hitherto inaccessible by conventional genetic/biochemical means. Finally, we also caution against solely relying on chemical-biology strategies and urge the field to undertake orthogonal validations to ensure robustness of results.

This article is intended to be a primer for the use of chemical biology. We focus on processes that are limited kinetically by reactive chemistry or that use reactive short-lived molecules to perturb and/or monitor individual-protein- or locale-specific function in living systems. We begin by discussing the need for chemical biology and the underlying design/execution of chemical-biology experiments, including ways to avoid pitfalls. We subsequently highlight some of the latest, and what we consider most interesting, chemical-biology approaches and evaluate their benefits and limitations. These methods are contrasted against classical genetic and chemical/biochemical techniques.

## The Need for Chemical Biology: *Beyond Genetics and Biochemistry*

Chemical biology occupies a niche that is not adequately filled by traditional biological sciences. Biochemistry/enzymology are suited to understand proteins in isolation, or in lysates. Using these methods, functions of individual proteins have been divined (Knowles and Albery, 1977), rates of specific steps of enzyme-catalyzed reactions have been elucidated, and development of tools to modulate a specific enzymatic process has been established. For instance, inhibition experiments directly impact physiological studies because inhibitors can downregulate specific enzymatic function expediently. Such events can in turn impact cellular pathways/processes rapidly, without the system being given the chance to compensate for the signaling changes incurred upon activity loss (as can occur during genetics experiments, *vide infra*). By measuring time-dependent effects, one can observe how the system responds to loss of the target protein's function. Such inhibition studies are of course also directly applicable to drug design and discovery. Perhaps Daniel Bovet's award of the 1957 Nobel Prize for physiology or medicine is the best example of the use of inhibitors for both academic and industrial pursuits^1^. The Nobel Prize in Physiology or Medicine 1957 was awarded to Daniel Bovet “for his discoveries relating to synthetic compounds that inhibit the action of certain body substances, and especially their action on the vascular system and the skeletal muscles.” Inhibitors continue to be used to study age- or context-specific loss of protein function for comparison to genetic knockout studies or to better model diseases (Ogasawara et al., 2018). Of equal importance, since knockout and chemical inhibition are not necessarily the same in terms of percentage of loss of function and the effect on global protein function, such chemical-biology experiments require careful consideration and data interpretation (Hedstrom, 2017).

Biochemistry/enzymology also are often used to study *robust complexes or* individual protein function, such as by immunoprecipitation, or activity assays. However, these experiments are typically carried out in lysates or on purified proteins. Cellular compartmentalization is lost under these conditions, and several other contextual factors are also perturbed. Such biochemistry experiments were instrumental in unraveling many fundamental processes, like the nature of triplet codons (Matthaei et al., 1962). But in many instances, the loss of context that occurs upon lysis or upon isolation incurs artifacts or loss of activity/structure, due to, for example, incorrect preparation techniques (Darling and Reid, 1979; Wang et al., 2011), or expression conditions (Osz-Papai et al., 2015). Furthermore, these assays also require large amounts of protein, thereby losing track of information on cell-to-cell variation, for example. Small-molecule bulk-exposure regimens tend to lack resolution at the sub-cellular and organ/tissue scales, unless cell-surface behaviors are investigated using cell-impermeable molecules, or deploying, as we see below, chemical-biological tricks. It is worth noting that the intracellular concentration of a small molecule is not necessarily the concentration of the molecule in the media. Especially for reactive small molecules (Liu et al., 2019), there is often a concentration gradient across the cell.

On the other hand, genetic tools have proven powerful for studying the functions and necessities of genes, pathways, and specific-protein functions. Such studies can often be carried out in the subcellular locale or organ/tissue of choice. High-throughput screening approaches to find specific genetic associations, that are difficult to identify otherwise, have been applied to global analysis of protein stability (Yen et al., 2008), cell-to-cell variation (Livet et al., 2007), and pathway intersections. Genetics—often guided through biochemical/structural studies, random mutation of putatively-important mutants, or genomic sequencing of selected mutants showing resistance to an inhibitor, or rescue of a specific phenotype—offers ways to manipulate *steady-state* information flow. Oftentimes, these experiments can be carried out at the single-cell level. However, genetic techniques often lack dynamic range and precise temporal control, especially in higher eukaryotes, in all but a few instances. Thus, new methods were required to deliver high-resolution information on transient associations, to enable investigations of rapid gain of function, to effect localized perturbation, or to zoom-in on the signaling behavior of individual proteins/pathways.

These questions above have all been tackled to varying success by chemical biology. Chemical biology offers the ability to generate reactive chemical signals at will, or modulate chemical properties of specific proteins at a preordained time, often in specific locales for a specific duration. In many of the most pertinent and informative scenarios, chemical-biology methods offer insight because they can generate “on demand” highly-reactive small molecules, whose half-lives and diffusion distances are short (Parvez et al., 2018). Localization or duration control are often achieved in conjunction with genetically-encodable elements that can serve as frameworks for biocompatible processes. Thus, although a simplification, chemical biology exists to some extent to: (1) bring the power of *in vitro* analysis to the cell, and ideally to the whole organism; and to (2) extend genetically-encodable functions beyond those accessible through the use of canonical amino acids. Indeed, much of the advances of chemical biology have been made by researchers that seek to perform work on questions at a “triple point,” i.e., the intersection of multiple fields, such as chemistry, enzymology, and cell signaling; or enzymology, bioinorganic chemistry, and genetics.

As chemical biology straddles several spheres of life sciences, we begin by discussing some intrinsic issues with chemical biology, and how they can be limited. We further discuss pitfalls and how to surmount them and aspects of good experimental design.

## Utility to Model Organisms

One key benefit of genetic methods/analysis is applicability to model organisms and humans (e.g., through heredity maps, lineage information). Indeed, simply examining if a mutant or disease is dominant vs. recessive, and other simple hereditary patterns, can give clues to disease mechanisms that are informative (Wilkie, 1994). Most enzymology/biochemistry techniques are less applicable to model-organism studies. Ultimately many inhibitors/small-molecule-probes are either too toxic, metabolically unstable, or administered in cell/tissue-penetrable “prodrug” forms, yielding limited information to link a precise chemotype/target-engagement to phenotype. Of course, there are some excellent small-molecule modulators/probes applicable to numerous model organisms. Indeed, 92% of approved drugs targeting the human proteome are small molecule-derived as opposed to biologics (Santos et al., 2017). Ideal chemical-biology methods should be applicable to model organisms; however, at the moment, few are generally applicable to much beyond cell culture. This limitation is in part because many methods require non-biocompatible chemical manipulations and/or use reagents either too toxic and/or impermeable to live models on the order of the experiment, thereby limiting the experiments to cultured cells or isolated organelles. Studies in lysates also remain a go-to strategy in proof-of-concept chemical-biology methods development, although these conditions provide little or no information on subcellular regulation or reflect close-to “real-world” conditions with respect to intracellular concentrations/assemblies/activities of specific macromolecules or metabolites. It is our hope that more model organism-based investigations with precise control in space, time, and context will surface in the future.

## Defining On-target Specificity: Necessitating Orthogonal Validations

Generally, a small molecule used for chemical biology should be as non-invasive as possible. Thus, it should not ideally cause cell death, cell-cycle stall, or adversely affect relevant pathways. Thus, changes in the aforementioned parameters induced by the small molecule should all be assessed. Such assessments should be made early in methods development. Since most cells in culture are grown in high-serum media, ideally the compound should not interact strongly with serum. Should serum be an issue, low-serum media is available, or cells can be switched to serum-free conditions during compound exposure. Ideally, in model organisms, the molecule should also not affect growth, development, or fecundity, among other easily-measurable parameters. Should a molecule/regimen satisfy the above criteria, one can consider it biocompatible. However, it should be noted that most genes are in excess and are not absolutely required for growth/survival, especially in the rich conditions that we culture cells and organisms. These rich conditions may mask negative effects that could be observed under the intended experimental conditions, or when other stresses are added to the system. This is an ongoing issue for all research that is not easily addressable. We will elaborate on some ways chemical biologists have practically obviated these issues below.

Based on our current understanding of the number of proteins, protein-modified states, and protein-protein/protein–nucleic acid interactions, as well as the sheer number of non-protein molecules in cells, it seems highly unlikely that any molecule is “100% selective.” Thus, there should always be concerns levied regarding off-target or artefactual effects arising due to use of small molecules, regardless of methods used to evaluate binding promiscuity 1962; Feldwisch-Drentrup, 2017. How binding promiscuity could impact a chemical-biology process depends on what the intended measurements are. Furthermore, observation of binding/labeling alone does not necessitate changes in enzyme activity/protein function, protein—protein/nucleic-acid interactions, or any process relevant to the intended experiment. Since the scope of this review does not warrant an extended discussion on IDing small-molecule targets, we focus here on methods aimed at assessing to what extent a specific output measured following a small-molecule treatment can be ascribed to a specific protein under study.

### Integrating siRNA/shRNA-Knockdowns to Small-Molecule Experiments

**Figure 1:**
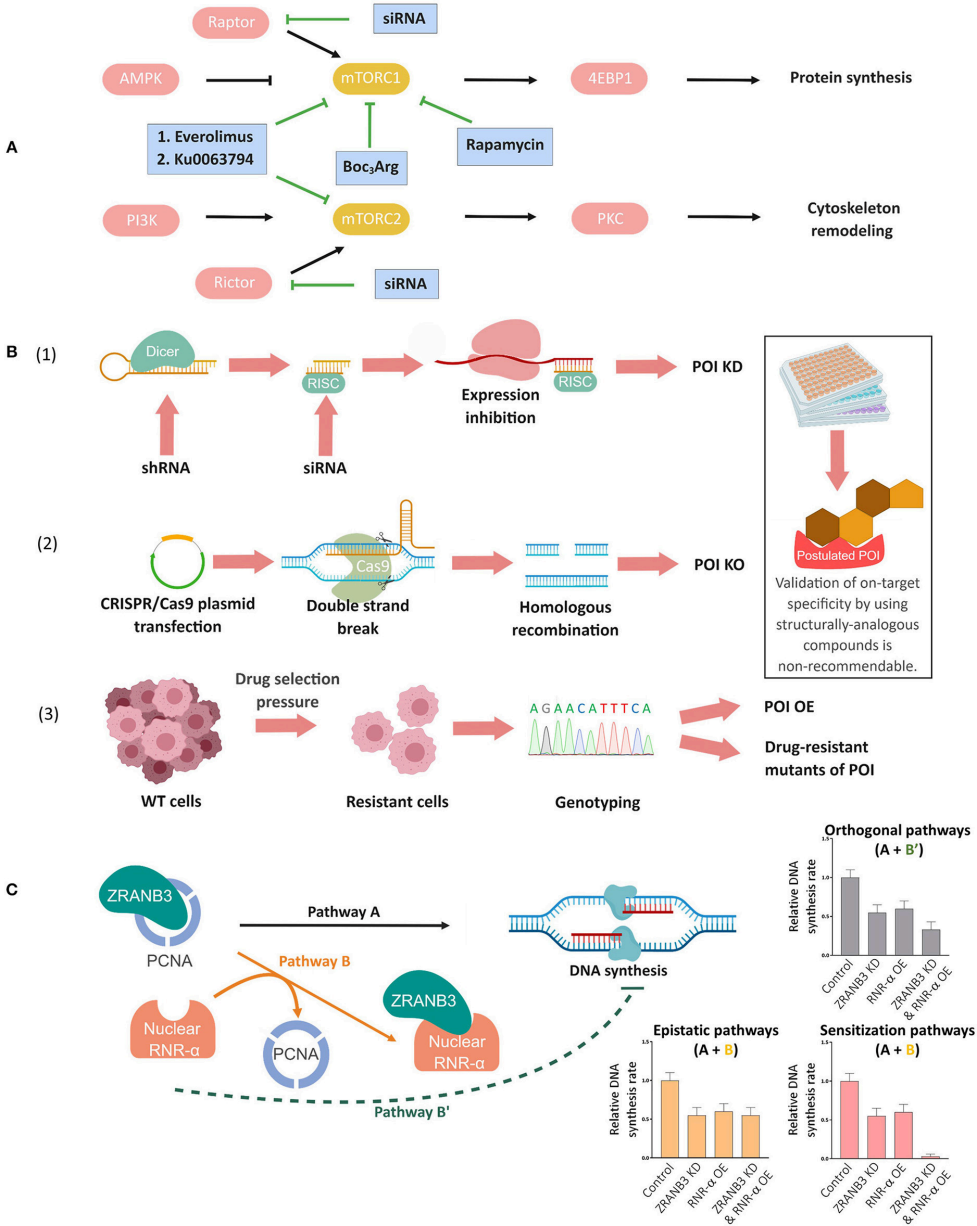
Complementary genetic methods to validate chemical biology outcomes. **(A)** Deconvolution of mammalian target of rapamycin complex (mTORC)-1 and-2 signaling pathways using mTORC-1 selective small-molecule inhibitors, dual inhibitors of mTORC-1 and-2, and specific knockdown of the respective companion (Raptor or Rictor) of each mTORC (Conciatori et al., 2018). **(B)** Orthogonal validations to confirm on-target specificity of small-molecule probes/inhibitors. (1) siRNA/shRNA knockdown (KD), (2) targeted knockout (KO) through CRISPR/Cas9 or other gene-editing techniques, and (3) genotyping in resistant cell lines. In (1), Dicer, a ribonuclease-(RNase)-III converts shRNAs into siRNAs. The cleaved double-stranded RNA is incorporated into the RNA-induced silencing complex (RISC) and the resulting strand complementary to mRNA subsequently inhibits mRNA expression. In (2), mammalian cells are transfected with a plasmid containing desired sgRNA/Cas-9 combination, which results in the formation of a ribonucleoprotein complex of the Cas9 protein and the sgRNA upon expression. Upon the complementation between sgRNA and the target sequence, the Cas9 protein undergoes allosteric activation and cleaves the double-stranded DNA. This double-strand break (DSB) will lead to either non-homologous end joining (not shown, but the most commonly employed strategy to make a genetic knockout) or homologous recombination with an ectopic DNA repair template containing a deletion sequence (shown in figure). Upon cell colony selection, the desired KO will be confirmed experimentally. In (3) continued exposure, typically to escalating concentrations of a drug, can lead to selection of resistant cells that may overexpress the target protein (POI OE), or express drug resistant mutants, amongst other possibilities discussed in the text and elsewhere. Inset: Note that on-target validation using different analogs of the compounds is non-recommendable (see text). **(C)** *Concept of epistasis exemplified by nuclear RNR-*α *and ZRANB3 in DNA synthesis*. RNR-α, once inside nucleus, binds to ZRANB3 nuclear protein, displacing ZRANB3's cognate binding partner, PCNA, *in vitro* and in cell lysates. But this data alone does not prove that such a mechanism occurs in an intact cell. Cells deficient of ZRANB3 (i.e., ZRANB3-KD) suppresses DNA-synthesis by ~30−40%, supporting the previous data on ZRANB3—PCNA binding-dependent DNA-synthesis (Pathway A). Overexpression (OE) of nuclear RNR-α suppresses DNA-synthesis to a similar extent but this result does not prove that ZRANB3 is a target of nuclear RNR-α in DNA-synthesis downregulation. By examining how nuclear RNR-α affects DNA-synthesis in ZRANB3-KD cells, the requirement of ZRANB3 for nuclear-RNR-α-dependent effects on DNA synthesis can be assessed. Indeed, in the absence of ZRANB3, nuclear-RNR-α-promoted DNA-synthesis suppression is ablated, supporting Pathway B (epistatic regulation via ZRANB3) over Pathway B'(direct downregulation, independent of ZRANB3), and also indicating that ZRANB3 is only a promoter of DNA synthesis (otherwise, the combination treatment would likely lead to a synergistic suppression of DNA synthesis). This analysis was also backed up by the fact that expression of RNR-α-binding-defective but otherwise functional ZRANB3-mutants renders cells resistant to nuclear-RNR-α-driven DNA-synthesis inhibition (Fu et al., 2018).

One of the most common ways to use small molecules for mechanistic analysis is to assess whether a specific protein's activity is required for a process. Proteasome inhibitors are, for instance, commonly used to investigate degradation mechanisms (Goldberg, 2012). When using small-molecule inhibitors that are well characterized to validate if the activity of protein of interest (POI) or pathway is required for a specific function, it is responsible to assay more than one inhibitor targeting the POI or pathway (Zhang et al., 2013; Coffey et al., 2016; Conciatori et al., 2018; Smith et al., 2019) (Figure 1A). The use of inactive structural/regioisomeric analogs of a single inhibitor to verify “on-target” effects is inadequate, since if a modification manifested within the analog silences an on-target binding event, it may also silence off-target binding events, or affect permeation/stability, and overall negative data could be misinterpreted as “proving” on-target specificity (Figure 1B). However, in cases where there is high enantiospecificity shown by a chiral ligand, inactive epimers of the inhibitor that are inactive are useful (Bondeson et al., 2015).

An alternative way to validate inhibition assays is siRNA, or knockout technologies. Knockdown/knockout and inhibition are not necessarily identical to small molecules in terms of overall effect (Weiss et al., 2007). However, if the POI's chemical activity is required for a specific process, the effects of knockdown/knockout and inhibition should often be similar. To rule out off-target effects of the siRNA, at least two siRNAs are best deployed, separately per experiment. If all conditions, or the majority of conditions, agree with the postulate, this is good evidence that the POI is involved in the pathway. The logic of such experiments runs that the off-target binding of structurally-dissimilar inhibitors, or sequence-dissimilar siRNAs, is unlikely to converge at a point other than the desired target. As the number of different components of the comparison, and indeed the chemical difference across the different comparisons, increases, the robustness of such a conclusion also increases.

Finally, we note that cell-line validation is critical for genetic processes. This process must include both antibody validation and functional validation of knockdown, but may need to be extended to genotyping genes of interest, especially in cultured cells (Long et al., 2017a). Rigorous literature searches are also helpful to uncover other known issues surrounding generated/evolved lines (Princiotta et al., 2001), or species-specific effects (Gupta et al., 1986) that can potentially cause confounding results.

### Integrating Resistant Cell Lines

If the mode of action of a compound is unknown, target-specificity validations are not as simple. One classic method to assess on-target effects of toxic drugs is to develop resistant cell lines, as these may overexpress the drug target (Figure 1B). Thus, there are known lines resistant to inhibitors such as hydroxyurea, MK2206, and methotrexate overexpressing their target protein, namely, ribonucleotide reductase(RNR)-subunit-β (Eriksson et al., 1984; Aye et al., 2015), Akt3 (Stottrup et al., 2016), and dihydrofolate reductase (Schimke, 1988), respectively.

However, it should be noted that there are multiple mechanisms to effect resistance, aside from direct upregulation of the target. Examining methotrexate resistance as an example, beyond target-gene upregulation, upregulation of efflux and altered drug metabolism are common routes to resistance (Bertino et al., 1996; Ercikan-Abali et al., 1997; van der Heijden et al., 2007). Indeed, transcription and translation of specific genes are often responsive to their products inhibition, meaning that cells actively work to countermand suppression of activity, and also that protein-upregulation often accompanies small-molecule inhibition. Thus, assessment of protein expression of the intended target following inhibition should be routine.

Protein overexpression itself can have unexpected consequences. An overexpressed protein can achieve micro-molar concentrations in mammalian cells (Zhao et al., 2018). Thus, particularly in instances where binding efficacy is moderate-to-high (a situation that can easily render concentrations of compound administered similar to, or significantly lower than, the overexpressed protein), the overexpressed protein could significantly reduce concentrations of active compound, sacrificially protecting other important targets, without it actually ever being a biologically-relevant target. Furthermore, protein overexpression can rewire signaling networks such that a drug may no longer be effective due to hyper-stimulation of a compensatory pathway.

### Integrating Functional Mutants

In some instances, overexpression of a resistant mutant—ideally with kinetic properties similar to that of the wild-type-protein—can also occur upon drug exposure. For instance, the discovery of RNR-α(D57N) mutant of the enzyme RNR that has similar *in vitro* kinetic activity (Aye and Stubbe, 2011) but is not inhibited by the native nucleotide dATP was discovered through such experiments (Ullman et al., 1980; Weinberg et al., 1981; Caras and Martin, 1988). These mutants can also be used to evaluate on-target specificity through overexpression, or better yet, through close-to-endogenous expression (Figure 1B; Wisitpitthaya et al., 2016). Under these conditions, mass action effects discussed above are much less likely, rendering the conclusions more incontestable.

### Integrating Epistasis Concepts

Modern approaches toward on-target specificity have focused on knockdown or knockout of the postulated target. Knockdown of a target typically sensitizes cells to a drug, because one can consider that some of the drug's “work” has been done for it, so it is easier for the drug to take effect. This sort of sensitization is most-commonly observed under conditions where there is little phenotypic output due to the knockdown. In some instances, knockdown of the protein is so acute that there is essentially a knockout of protein function. Under these conditions (or with true knockouts), one may expect there to be essentially no impact of the drug, as there is no target. This condition is termed *epistasis* (Cordell, 2002; Miko, 2008). If the drug functions through processes not associated with the proposed target, then similar fold effects will still be seen in the knockdown/knockout line. This sort of analysis applies equally to protein-based inhibitors and small molecules.

Our laboratory recently used such arguments to elucidate the pathway through which nuclear-translocated RNR-subunit-α suppresses DNA synthesis (Fu et al., 2018; Figure 1C). Our data showed that RNR-α is able to bind to a nuclear-localized protein called ZRANB3. We hypothesized that this interaction may inhibit ZRANB3-function because ZRANB3—RNR-α interaction also led to disruption of PCNA binding to ZRANB3, and it is known that the ZRANB3—PCNA complex plays a role in DNA damage response (Poole and Cortez, 2017). Our data revealed that robust (~85%) knockdown of ZRANB3 by three different siRNAs suppressed DNA-synthesis rate by 30–40% (Fu et al., 2018), showing that ZRANB3 is a promoter (although not necessarily required) for DNA synthesis, and that in the knockdown states, ZRANB3's activity is significantly depleted. Overexpression of RNR-α (which raises nuclear RNR-α levels, allowing ZRANB3 to bind RNR-α) also suppressed DNA-synthesis rate. Thus, there were several possible scenarios: (assuming ZRANB3 were a promoter of DNA synthesis and RNR-α inhibits ZRANB3) we would see that ZRANB3-knockdown cells (that have significantly lost ZRANB3's DNA synthesis promoting function) are resistant to RNR-α overexpression; (assuming ZRANB3 were *required* for DNA synthesis and RNR-α inhibits ZRANB3) we would see a large fold increase in fold suppression of DNA synthesis upon RNR-α overexpression in the knockdown lines as the minimal ZRANB3 remaining would be overwhelmed by the influx of the inhibitor RNR-α; (assuming ZRANB3 does not mediate the nuclear-RNR-α-promoted DNA-synthesis suppression) there would be a drop in DNA-synthesis rate when RNR-α was overexpressed in the ZRANB3-knockdown cells of the same fold to what was seen in the control cells. The first of these outcomes was observed (Fu et al., 2018), consistent with nuclear-RNR-α acting as an inhibitor of ZRANB3-function and ZRANB3 being a promoter of DNA synthesis. We validated these experiments by showing similar outcomes upon dATP treatment, a situation that causes RNR-α to translocate into the nucleus. We were also able to derive a functional point mutant of ZRANB3 (that cannot bind RNR-α but can promote DNA synthesis), and this mutant was resistant to RNR-α-overexpression (Fu et al., 2018).

Of course, one must be careful to interpret how knockdown, and especially knockout, affects cells. For instance, in some cases even 85% knockdown of the target protein is not sufficient to observe significant pathway flux change (Lew and Tolan, 2012), thus it may not be possible to obtain significant sensitization using siRNA. Furthermore, since knockout of a target protein should ideally mimic saturating drug behavior, knockout may not be tolerated. Thus derived knockout lines can suffer from change of flux through necessary pathways, likely suppressing growth rates, or selecting for cells with modified survival responses, etc. Such residual knockout cells can appear resistant, solely because a few outliers from the population have been selected. However, the observance of resistance in knockout lines still remains good evidence for an on-target mechanism (Chauhan et al., 2012). Accordingly, the use/derivation of resistant point mutants as performed above are highly useful for mechanistic studies.

## Dealing With Ectopic Proteins: Judicious Choices for Minimal Interference

Aside from employing ectopic small molecules, many chemical-biology methods employ unnatural (often fusion) proteins ectopically expressed in the system under study. The effect of these non-native elements must be considered when evaluating each specific method and the data each method produces. Any perturbation imposed by the ectopic protein on the basal levels of pathway signal and the responsivity of the pathway must be assessed.

### Transient Expression vs. Stable Integration

**Figure 2:**
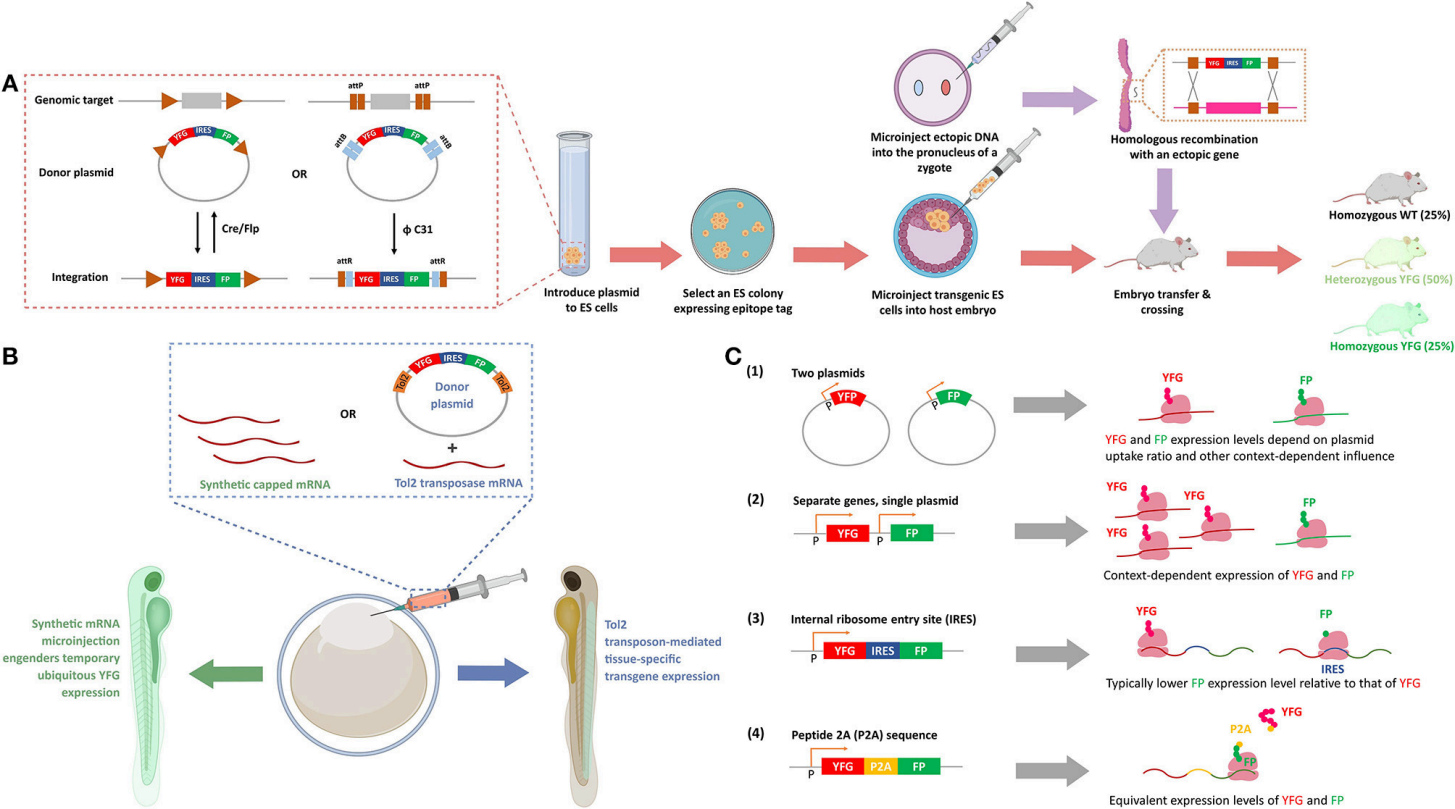
Methods to express ectopic protein through introduction of ectopic nucleic acids. **(A)** Genetic integration of POI in cell lines and transgenic (Tg) mice. Inset: The recombinases [Cre and Flipase (Flp)] can be used to exchange genomic DNA with plasmid DNA to promote targeted integration of your favorite gene (YFG) into specific cell lines, or a suitable host. The bacteriophage ΦC31 integrase can perform a similar function in mouse. ΦC31 enables site-specific integration of the genes flanking phage attB-sites into the locus of host genome that is pre-engineered with up- and down-stream phage attP-sites. This recombination is irreversible because the resultant attR-flanked genes in host genome is not recognized by ΦC31 integrase. (FP, fluorescence protein; IRES, internal ribosomal entry site, that enables bicistronic expression of, in this figure, YFG and FP). To create a Tg mouse, a suitable embryonic stem (ES) cell line is prepared, and post integration of YFG-IRES-FP, colonies that have undergone insertion are selected and subsequently injected into a fertilized cell, giving rise to mosaic progeny that can be further manipulated. After multiple crossing with wild-type (WT) mice, 25% and 50% of the progeny have homozygous and heterozygous YFG knock-in, respectively (lower row, arrows in salmon). Alternatively, DNA with homology arms overlapping with the target site of interest can be injected into the pro-nuclei of fertilized embryos, and post-recombination, targeted-knock-in of the specific allele is created (top row, arrows in purple). **(B)** Transient expression and stable integration of POI in zebrafish. The eggs of zebrafish, which undergo external fertilization, can be injected with synthetic mRNA, to give ubiquitous, transient expression of YFG. Alternatively, a number of other random- or targeted-insertion protocols can be used. In this case, the transposon Tol2 is shown that gives random-integration of YFG—IRES—FP construct. Depending on the promoter driving the transgene (here, YFP and FP), locale-specific or ubiquitous expression can be achieved. **(C)** Different methods of POI expression in cultured cells. (1) Transfection of two plasmids gives a population of cells that express both genes (YFG and FP), with expression levels of each gene varying widely from cell to cell, with little correlation in the relative expression levels of each gene. (2) Separate genes on single plasmids can give standardized amounts of each protein, although the ratio of each protein is context-dependent, as transcription, mRNA stability and translation are independent between each protein (YFG and FP). (3) In IRES-driven bicistronic expression systems, transcription and mRNA stability of each protein are the same, but translation of the two genes (YFG and FP) can be considerably different. Typically, the protein downstream to IRES is expressed to a lower level than the one upstream to IRES. (4) In P2A-driven systems, transcription, mRNA stability, and translation are all the same (see text for details). (P designates promoter-binding site in **B,C**).

Considerations should also be made on how such transgenes will be introduced (Figure 2A). For cell culture, transient transfection is common. This procedure tends to give high levels of expression that are significantly variable across individual cells. Thus, single-cell analysis must be performed carefully such that expression of transgene is normalized, or at least accounted for. Furthermore, bulk phenotypic outputs could be derived from a subset of cells from the transfected pool (Parvez et al., 2016). Transfection also requires large amounts of plasmid, and chemical treatment of cells with lipid or other reagents.

An alternative is the use of cells containing integrated copies of the plasmid, or integrated ectopic DNA. Integration can be achieved under conditions of prolonged selection post transfection (Lin et al., 2015), through viral integration, or through transposases, all of which typically give “random” incorporation usually at multiple loci. Targeted integration (which limits chances of integration incurring off-target effects) using FLIP-recombinase (Schlake and Bode, 1994; Fu et al., 2018) or similar setups has also become popular (Figure 2A). Overall benefits of integration approaches include: obviating the need to transfect, which gives increased reliability, and reduced variations across experiments. Furthermore, single clones of lines can be chosen to ensure uniform expression of the transgene. If several clones are picked, a range of expressions can be chosen. Alternatively, inducible lines can be used that give calibrated expression.

Model organisms vary in their analogies to cell culture in these respects. Some are routinely manipulated transiently or through modification at the genome level during experiments. Zebrafish is a good example of such a system (Figure 2B). Others are almost exclusively manipulated through heritable manipulations, such as *C. elegans*. There are also some species-specific quirks, such as the ability of worms to form stable extrachromosomal arrays. We have shown that this system can prove very useful for chemical biology as the array-containing worms can be selected using a visual marker (e.g., fluorescence), and give a predictable percentage of transgenic progeny vs. wildtype progeny (Hall-Beauvais et al., 2018; Long et al., 2018a). Thus, experiments ultimately contain both wildtype and transgenic animals derived from the same founders that have been exposed to identical experimental conditions.

### Knock-Out/Knock-In Lines

There are several issues that need to be considered when planning to derive knockout or knock-in lines. There are important differences between implementing these approaches in cancer cell lines vs. model organisms. Indeed, the aneuploidy of cancer cells renders knock-in generation difficult to achieve currently. Some cancer cells lines are “near diploid,” such as HCT116, which has enabled homozygous and heterozygous knock-ins to be made by several methods we describe below (Duncan et al., 2012). The issue of aneuploidy in cancer cell lines could also potentially contribute to difficulty in generating knockout lines in cancer cells, although this does not appear to be a huge factor for CRISPR-Cas9 technologies (Yuen et al., 2017). Notably, the on- vs. off-target effects of all genetic manipulation strategies are hotly debated (Gallagher and Haber, 2018; Wang et al., 2018). These issues in whole organisms can be overcome by outcrossing, but this issue is not possible to “fix” in cultured cells.

In general, targeted genome modification involves introducing a specific targeted DNA double-stranded break (DSB), which is then fixed either by homologous recombination (HR) or through non-homologous end joining (NHEJ). Indeed, the ability to induce specific DSBs is a critical factor of these experiments, explaining why targeted nucleases are such “big news.” DSB can be achieved by expression of a nuclease that will specifically cut at an intended locus. In the modern era, DSB is most-readily introduced using CRISPR-Cas9, although other nucleases, including TALENs (Joung and Sander, 2013), have been and are still used. If there is no DNA with which to undergo recombination, the cell can repair the damage through NHEJ. This is an error-prone method that leads to formation of an “indel” (insertion/deletion polymorphism). Ideally, the indel creates a premature stop codon in the protein of interest (POI), yielding a truncated POI, although clearly frameshifts often also occur. It is important to appreciate that such systems create a “non-functional” protein, although typically mRNA-production still occurs and a gene product is often still generated, which could retain some bioactivity.

Knock-in is a particularly useful method to study the consequences of unnatural gene-products. Classic methods involve injection of linearized ectopic DNA into pro-nuclei of zygotes, and rely upon spontaneous HR (an error-free process) to introduce the gene of interest. Often times, the ectopic DNA contains a selection cassette, which can be removed post selection by standard methods. In more modern approaches, a DSB is induced consequentially with the introduction of the DNA (either short single-stranded DNA with short overlapping regions, or longer double-stranded DNA) to promote recombination. Thus, the ectopic DNA can be incorporated into the locus of the break more efficiently.

Recently, CRISPR-Cas9 has become a method of choice for generation of DSBs to assist in generation of knock-in lines (Platt et al., 2014), in a range of organisms including mice (Singh et al., 2015), worms (Dickinson and Goldstein, 2016), and parasites (Cui and Yu, 2016). This requires the expression of Cas9, which can be achieved through the injection of protein, DNA, or mRNA. Although numerous factors intrinsic to the protein affect protein expression (such as protein stability), typically, a knock-in line is the most likely to lead to a set-up where protein expression (and protein translational/transcriptional regulation) are unperturbed compared to wild type. Unfortunately, as alluded to above, we currently lack the ability to generate knock-in lines of most cancer cells. Thus, knock-in cell lines are derived from genetically-engineered organisms, whose cells are harvested and then adapted into cell lines. This approach can work well, even if the knock-in is unable to survive to adulthood, as all that is required are relatively early embryos (Fu et al., 2018).

Cre-Lox has been used to perform context- or stage-specific manipulation of numerous organisms (Figure 2A), and similar concepts using CRISPR-Cas9 are also being introduced (Katigbak et al., 2018). Critically, CRISPR-Cas9 editing can be conducted in adult mice, under correct conditions. Such strategies are particularly relevant to knockout/mutation of essential genes and generation of targeted disease models.

### Fluorescent Proteins and Epitope Tags

Regardless of the model system and expression system, ideally, all non-canonical/ectopic sequences, or protein domains should be removed from the expressed POI. For instance, it is common to express ectopic POIs as fusions with fluorescent proteins (FPs) or other chemically reactive domains, like Halo or SNAP, etc. Our subsequent discussion focuses on FPs, which are arguably most commonly used and for which several issues have been raised. However, protein-specific caveats likely apply to all ectopic protein domains and to a lesser extend linker regions and epitope tags. Efforts to assess validity (e.g. activity, localization, response)/rule out artifacts (unexpected effects on critical pathways, unexpected growth defects, etc.) in the specific system of use should always be made. FPs are certainly not bio-inert (Koelsch et al., 2013; Coumans et al., 2014; Ansari et al., 2016; Ganini et al., 2017); their use should be restricted where possible, especially in cell culture, unless, for instance, single live cell analysis is required. If FPs are required, whenever possible, fusion of FP to the POI can be circumvented through the use of separate promoters, plasmids, or, more reliably, through the use of bicistronic systems, such as IRES, or self-splicing peptides, like P2A. IRES/P2A also allow relative protein expression to be assessed across different cells (Jang et al., 1988; Pelletier and Sonenberg, 1988; Trichas et al., 2008; Figure 2C). It is important to note that IRES and P2A, are cell-type and organism dependent in terms of their effectiveness. Furthermore, P2A gives similar levels of each protein post translation (assuming each protein has similar stabilities Liu et al., 2017), but IRES biases expression against the protein downstream of the IRES (Mizuguchi et al., 2000). These nuances have been judiciously employed in experimental designs (Wang et al., 2015). The excitation and emission wavelength of the FP should be chosen to be as far away from background fluorescence and from any other photochemistry/redox-chemistry that is being conducted. Typically, red fluorescence gives better signal to noise.

In model organisms, such as *C. elegans* and *D. melanogaster*, the use of FPs as reporters of transgenicity are common, although dominant phenotypic markers, such as *rol-6* (“roller”) or *ro*+ (“rough”), are also used (Lockett et al., 1992). These phenotypic markers tend to be derived from known dominant mutants, leading to obvious physical deformities, and hence are “hard-wired,” and as they affect, for instance, motility, they may pose undesired impacts on physiology. By contrast, for FP's, the locale of expression can be defined by the user. One can consider restricting fluorescence to a small number of cells (e.g., touch neurons, via the *C. elegans* promoter *Mec7*, in worms), or to cells that are not intended for the specific chemical-biology experiments (such as pharynx expression, via the *C. elegans* promoter *Myo2*, and gut expression of the key transgene, via the *C. elegans* promoter, *Ges-1*). If it is necessary to mark the specific transgenic cells with FPs, IRES, or likely better P2A fusions of the required gene with an FP, are best deployed (Figure 2C).

Furthermore, directing subcellular localization of the FP to a region that is not intended to be studied can also be useful, such as fusing the FP to a nuclear-localization sequence (NLS), if studying cytosolic processes with the specific POI. Finally, expressing POIs with epitope tags (FLAG, HA, V5, etc.) is often useful in cell culture for enrichment of specific proteins, but it is almost essential in model organisms, especially those for which homology with humans is only moderate, e.g., *C. elegans*. If the POI is fused to another protein-tag, such as Halo-Tag, for which highly-specific, low cross-reactivity antibodies are available (*vide infra*), such antibodies can often substitute. However, the background labeling of the antibody for the proteome from the species of interest should be evaluated, prior to design of the fusion construct, and the requirements of the whole experiment should be considered.

### Additional Considerations

Ideally, activity/function of any fusion-POI expressed should be compared against the non-fused/non-tagged POI. This assessment can be done *in vitro*, or by measuring expected phenotypes/responses in cells/organisms in which the native protein has been depleted (by RNAi, or genetic knock out) and the fusion-POI exogenously supplemented. Knock-in lines are ideal, especially when the gene in question is essential, although for most purposes, simultaneous knockdown of a specific gene and plasmid transfection is operationally similar (provided the knockdown leads to a measurable effect on the cells that can be rescued). In cases where simultaneous siRNA knockdown and re-expression of the modified target POI is performed, the ectopic POI typically should have a synonymous mutation of the siRNA target sequence. The re-expressed protein should be shown to occur at close to endogenous levels.

Because most chemical-biology approaches are multi-parameter, and these parameters often interact to make a new state that is not a simple “sum” of the individual parts, it can be argued that there is no “ideal” chemical-biology method. Hence, we emphasize that all chemical-biology approaches should be validated orthogonally. However, we also stress that close-to-ideal chemical-biology methods lend themselves to the development of as careful-and-as-close-to-“real” controls as possible. For our purposes, this means that assay conditions can be modulated such that *a single variable* is changed at a time (e.g., using a point mutant that does not process the chemistry/signal-propagation as intended). In fact, when such controls are available or built into the design, results tend to transpose well to real-life and any small-molecule-induced perturbations to the resting state of the cell can usually be tolerated.

## Perturbation Strategies: *Complementing Biosensors*

Most chemical-biology perturbation methods begin with the system at a “basal state” followed by a rapid jump to a new state induced by some form of chemical perturbation. The time taken to reach the new state/or the reactivity of the molecules generated, is often a key parameter that must be optimized such that genuine dynamics of cell responses can be measured on their relevant timescales. Thus, just as in classic kinetics experiments, the speed of the perturbation must be faster than other ensuing processes intended to be measured, so that a true readout of the chronology of the responses is established. In many instances, chemical-biology perturbations can elicit a shift to a “new state,” or generate a reactive small molecule in minutes or even seconds.

By contrast, the length of time required to synthesize RNA and protein, and indeed the half-lives of most proteins are typically hours (Vogel and Marcotte, 2012; Liu et al., 2016; Mathieson et al., 2018) in higher eukaryotes, although precise values are species- and protein-dependent. Indeed, genetic techniques, like LOX/tet/RNAi/Flip/CRISPR-Cas9, also have a long latency. Arguably the “fastest-responding” genetically-encodable element is modulation of protein degradation (either positive or negative). The fastest responding of these strategies are mostly chemical genetic in nature. Such perturbation methods include: SHIELD (Banaszynski et al., 2006), auxin-initiated decay (Nishimura et al., 2009), HyT (and derivative stress induction methods) (Neklesa et al., 2011), Boc3Arg (Shi et al., 2016) and PROTACs (Lai and Crews, 2017). There are also some systems based on heat-shock that can be applied to organisms such as yeast (Dohmen et al., 1994), but one must consider the pleiotropic effects of temperature on the system in these instances as well. Despite these methods being considered rapid, 1–3 h is still required to have a significant effect (assumed to occur around 50% protein depletion, although much greater degradation than that can be required Lew and Tolan, 2012), and the slow step is almost certainly the change in stability of the protein target, not the engagement of the small molecule with its protein target, or the thermal unfolding of the protein (further underlining the relative slowness of biological recognition processes; Shamir et al., 2016).

Recently, photoactivatable cell signaling has also become common. This overall strategy represents another rapid response genetic unit. Importantly, this strategy has proven applicable to multiple different biological problems (Zhang and Cui, 2015), often (although not always) where oligomerization, dimerization, or recruitment are required for changes of cellular protein activity. In many instances, the system is also reversible, rendering these systems ideal for measuring signal-induced changes. Another group of rapid genetically-encodable methods involves chemical-induced dimerization (Stanton et al., 2018), a strategy that dates back to the earliest years of chemical biology (Spencer et al., 1993), but has found applications to modern genetic approaches, such as split-Cas9 (Zetsche et al., 2015). Using Cas9 as a specific example, intended outputs (e.g., transcriptional regulation, gene “deletion” or DNA damage) occur post Cas9 binding to its target DNA-sequence, meaning they are inherently controlled by factors with relatively long latency, such as mRNA stability or recruitment of transcriptional apparatus. Furthermore, Cas9 target engagement can take hours (Jones et al., 2017), even in *E. coli*. Finally, genetically-encodable sensors: such as the ROS-sensors: HYPER (Belousov et al., 2006) and roGFP (Meyer and Dick, 2010), the calcium sensor: m-GECO (Zhao et al., 2011); as well as the fleet of biosensors for kinase-activity (Mo et al., 2017) or metabolite sensing (Litke et al., 2016; Tao et al., 2017), are also rapidly-responsive elements, although these are not perturbation/labeling tools. However, use of these established visualization/activity-monitoring methods in conjunction with perturbation strategies—such as inhibition, integration of dominant-negative or gain-of-function alleles, or other targeted chemical-biology perturbation approaches that we outline below—offers a gateway to study cell responsivity and pathway architecture.

## Parameters

In this section, we discuss a few representative chemical-biology techniques (chosen for illustrative purposes from the much larger armory of methods these days available to researchers) in terms of the following parameters: (1) occupancy, i.e., whether these techniques are intended to saturate their targets or label only a subset of available targets based on some parameter; (2) spatial restriction, i.e., how constrained the techniques can be to a specific macromolecule, organelle, or interactome; and (3) functional information, i.e., what can we learn about the consequences of localization/reactivity/labeling using the technique. We further discuss the overall invasiveness of the methods and controls that are built in to the methods and orthogonal validation of the data. We will also evaluate how compatible and comparable these methods are with genetic/biochemical techniques.

### Target Saturation Methods to Probe Protein Localization and Associations

These methods are not strictly direct perturbation methods. However, they ideally employ reactive small molecules, generated on demand to chemically tag a specific set of proteins present in a chosen locale, or interactome, at a specific time (Table 1). These tagging processes thus reveal proteins either localized within a specific region or associating with a specific protein, even if they are present in low amounts, or if the interactions occur for a short period of time. Optimal parameters for these methods typically involve minimizing diffusion distance of the reactive molecule (Parvez et al., 2018), restricting membrane permeability (Yang and Hinner, 2015), and controlling the exposure time of the reactive entity to the native environment (Long et al., 2017c; Parvez et al., 2018).

**Table 1:** Comparison of the representative chemical-biology techniques discussed in this review.

	**PROTAC**	**APEX**	**BioID**	**(mini) Turbo ID**	**si/shRNA knockdown^a^**	**T-REX**	**G-REX**
Effective time (*t*_1/2_)	1–3 h	1 min	~18 h	10 min−2 h	Variable (hours to days)	1 min	1 min
Occupancy	High	Proximity-dependent	Proximity-dependent	Proximity-dependent	Time-dependent	Target-dependent	Low
Spatio-temporal control	Low	High	Medium	Medium	Low	High	n.d.^b^
POI-specific functional information	High	Low	Low	Low	Variable (dependent on target intrinsic stability)	High	Low
Organismal applications	High	Low	Medium	Medium	Medium	Medium	Medium

a *RNAi is highly species dependent. It is extremely efficacious in worms, where effects can also be heritable. In higher eukaryotes, RNAi delivery is a significant challenge, although there are some drugs that have entered trials. Yeast and other lower eukaryotes and prokaryotes lack RNAi machinery*.

b *Not yet determined*.

#### APEX

**Figure 3:**
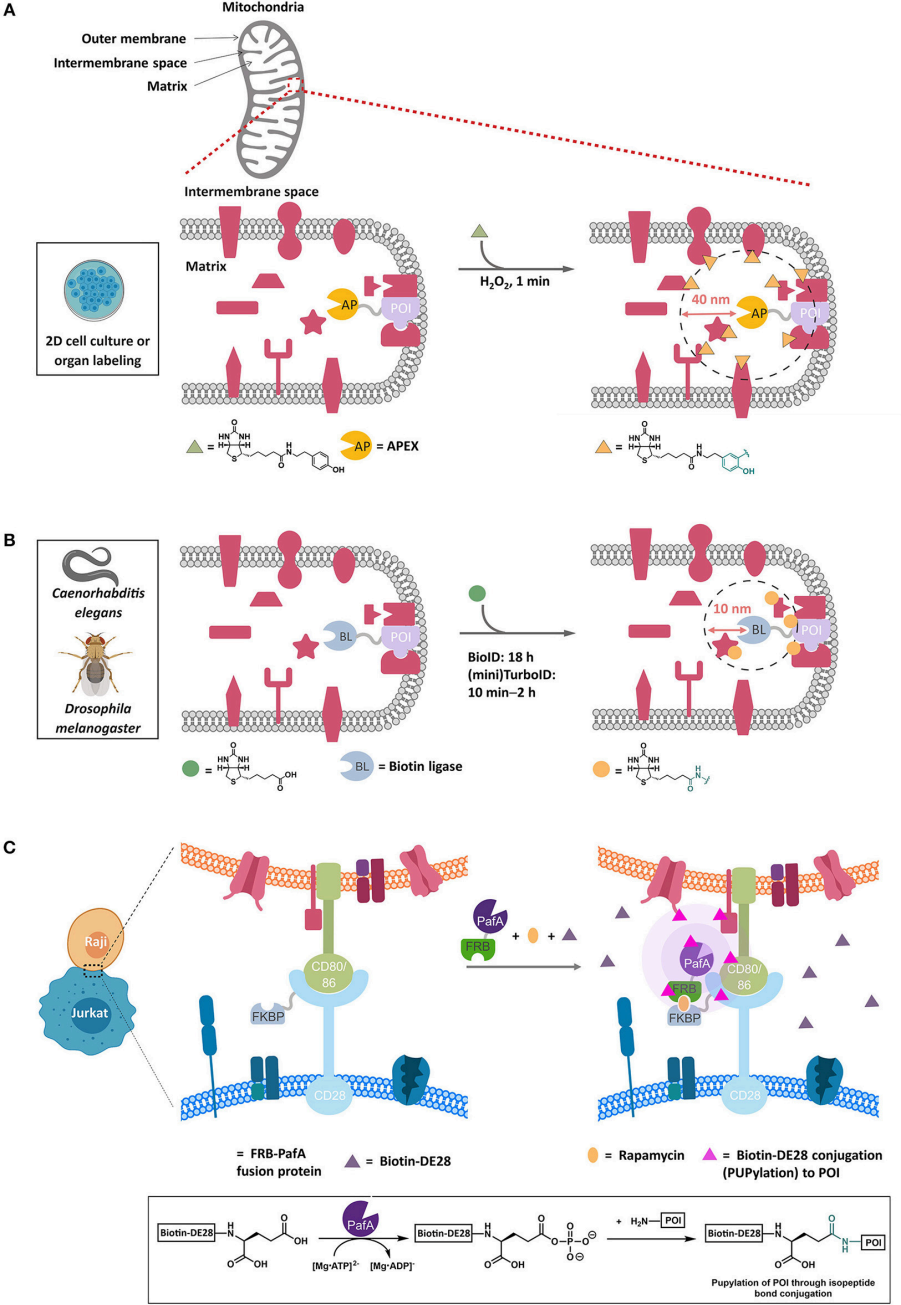
Representative proximity-based target ID platforms. **(A)** APEX method. APEX-fused POI is overexpressed in live cells and targeted to the compartment of interest, such as the mitochondrial intermembrane in this example. The typical experimental procedure involves incubating cells with biotin-phenol (30 min, 0.5 mM) followed by a brief exposure to H_2_O_2_ (1 min, 1 mM) to initiate the biotinylation of the proteins proximal to the POI (within ~40 nm). After quenching the oxidants, cell lysis and streptavidin enrichment, the biotinylated proteins can be ID'ed by trypsin-digest and LC-MS/MS analysis. Currently, APEX's application is limited to 2D-cell culture and isolated organs. **(B)** BioID method. In this proximity-biotinylation catalyzed by promiscuous biotin ligases (BLs), various forms of BirA mutants have been employed, with TurboID and miniTurboID as the latest, leading to biotinylation of proteins spatially close to the POI (fused to BL). These biotin ligases catalyze the formation of biotin-5′-AMP ester, which diffuses out of the active site and is captured by the accessible nucleophilic residues (primarily lysine) of the POI and those of the proteins within 10 nm on average from the POI. BioID experiment typically requires 18 h or longer periods of time to achieve significant biotinylation of the proteins. TurboID and miniTurboID have significantly improved the biotinylation efficiency (10 min – 2 h) with little difference in labeling output. TurboID and miniTurboID have been successfully applied to tissue-specific protein ID using *D. melanogaster* and *C. elegans* as model organisms, although the optimized biotinylation conditions of TurboID and miniTurbo in human cells may not be transposable to these organisms, owing to the differences in growth temperature and time scale. **(C)** PUP-IT method. This method IDs protein–protein interactions through proximity-PUPylation on cell surface; e.g., PUP-IT has been applied to mark the cell–cell recognition events between Raji and Jurkat cells. The FKBP fusion of CD28 is stably expressed on cell-surface membrane in Jurkat cells. Upon addition of rapamycin (orange oval) and PafA(PUP ligase)-FRB fusion protein to the growth media, a functional PUP-IT complex proximal to CD28 receptor is formed. The addition of biotin-DE28 (purple triangle)—the truncated PUP protein that remains active in PUPylation reaction catalyzed by PafA—enables PUPylation of Raji cell-surface proteins CD80 and CD86 rapidly (magenta triangle), which are known to interact with the CD28 receptor in Jurkat cells. Inset shows the underlying chemistry. FKBP, FK506 binding protein. FRB, FKBP-rapamycin binding domain.

APEX is an extremely useful method to profile protein localizations and associations. The key to this method is deployment of an engineered soybean ascorbate peroxidase protein that creates a reactive biotinylated phenoxyl radical, which has a short half-life (1 ms), giving APEX a labeling range of around 40 nm (Rhee et al., 2013; Figure 3A). The generation of this molecule within a defined compartment/region gives high resolution for assigning localizations and associations, which can be carried out in a multiplex manner (Cruz-Lopez et al., 2018). For instance, novel mitochondrial-associating proteins have been identified using APEX. Recent successes of APEX include profiling of associations at specific genomic loci (Myers et al., 2018) (through fusion to a binding-competent but catalytically-inactive mutant of Cas9, dCas9) and identifying new protein-protein associations (Xue et al., 2017). APEX has also recently been applied to RNA immuno-precipitation (Kaewsapsak et al., 2017). Thus, APEX was one of the most-widely used chemical biology methods in 2017–2018. Soybean peroxidase, from which both APEX-based probes are derived, is also known to be relatively robust to pH and temperature (Henriksen et al., 2001), although it does require a metallocofactor, and hence activity is dependent on correct loading of the cofactor, which could be context dependent.

Because of the short half-life and ectopic nature of the phenoxyradical (Rhee et al., 2013), APEX is unable to probe, for instance, downstream signaling. It is noteworthy that APEX outputs, although internally subject to not much more than 20% variability run to run, are subject to significant variability dependent upon the comparison used (Markmiller et al., 2018). Thus, multiple cross comparisons may be required to build up a complete picture of the interactome. Furthermore, APEX tends to favor labeling of unfolded proteins (Minde et al., 2018), so there is certainly the possibility for biased outcomes. Soybean peroxidase is known to generate spontaneous reactive oxygen species under certain conditions, and hence APEX could confer context specific changes to cellular redox levels that are difficult to address (Kimura and Kawano, 2015). How the ectopic overexpression of peroxidase may alter signaling/interactome architectures and redox homeostasis, etc., has not yet been assessed. Furthermore, APEX requires stimulation with peroxide (~1 mM) to form the phenoxy radical. This requirement likely restricts the use of this technique to 2D-cell culture/isolated organs (Chen et al., 2015), as it is unlikely whole organisms will take up peroxide equally. Peroxide treatment is only for a relatively short time (minutes), but even brief pulses of peroxide can alter signaling pathways, elicit translocation, and affect protein stability/integrity (Parvez et al., 2018). Many small molecules can also be inactivated/compromised by peroxide treatment. APEX has, however, proven to be compatible with some important methods, including EM (Martell et al., 2017; Mavlyutov et al., 2017). Control for the peroxide is an intrinsic challenge of the peroxidase-based platform. The bioactivity of the phenoxy-radical-precursor that cells are treated with for significant dose (0.5 mM) and exposure-time (0.5 h) prior to oxidation as well as the post-treatment quenching regimen (5 mM Trolox and 10 mM ascorbate performed 3 times), also remains untested.

#### BioID

BioID uses an engineered promiscuous *E. coli* biotin ligase. This protein generates a reactive biotinylated molecule, biotin-AMP, *in situ* (Figure 3B). Biotin-AMP is a type of acyl phosphate, which are short-lived species whose hydrolysis liberates around as much energy as ATP hydrolysis (Meyerhof and Shatas, 1952; Di Sabato and Jencks, 1961). Acylphosphates are kinetically less stable than ATP, although they are more stable than the phenoxy radical generated by APEX in water (Long et al., 2016). However, in the biological milieu, biotin-AMP is ephemeral and has a diffusion distance of around 10 nm (Kim et al., 2014) (i.e., is likely more spatially restricted than APEX and may ultimately give protein-level resolution Rees et al., 2015), and is capable of labeling proximal lysines. One critical difference between APEX and BioID is that BioID does not require stimulation with peroxide to generate the intermediate, and as such BioID has been applied to multiple model organisms including mice, *Toxoplasma gondi* (Long et al., 2018b), and slime molds. Recently, there have been some extensions to this method to improve the context dependence, including split-BioID (De Munter et al., 2017; Schopp et al., 2017). Critically, two different split proteins have been reported, testifying to the versatility of BioID. Finally, BioID has recently been coupled with affinity purification MS, using Strep-II tag to allow more quantitative analysis of interaction distances across large complexes (Liu et al., 2018b).

One of the major issues with BioID is the slow kinetics of formation of biotin-AMP, which can particularly restrict use in organisms that are not grown at 37°C. This issue was recently overcome by engineering ligases with heightened kinetic proficiencies. TurboID and miniTurbo (a truncated version of TurboID) allow substantial biotinylation of the proteome in a few hours, as opposed to the typical 18 h in BioID (Branon et al., 2018). Although, little difference in the labeled proteins detected was observed between the two conditions in cultured cells, several important applications were shown in model organisms. For instance, the embryonic development of *C. elegans* is approximately 14 h and its optimal development temperature is 16–20°C (Zhang et al., 2015). Thus, a rapid labeling strategy is required to enable sufficient build-up of labeled protein in the embryonic stages. As may have been predicted, BioID did not label embryos well, but TurboID and miniTurbo yielded robust labeling.

BioID requires ATP to generate the reactive species. Thus, one could consider BioID to be more cellular context-dependent than APEX. Although this question has not been systematically addressed, and despite ATP being a critical component of the cell, it is known that ATP levels are fluxional (Imamura et al., 2009; Tantama et al., 2013) and time-(Schneider and Gourse, 2004) and locale-dependent (Suzuki et al., 2015) and variable cell-to-cell (Yaginuma et al., 2014). Furthermore, although many of the issues of BioID were solved by Turbo/miniTurbo-ID, it is worth noting that these proteins are not inert and can potentially stress cells/deplete cellular resources. Evidence for this was provided as TurboID-expressing worms were developmentally delayed, although this was not observed in miniTurboID-expressing worms; the reasons for these differences are unknown. It is possible that split TurboID would obviate some of these issues.

#### Reactive Ubiquitin Analogs

Another recent innovation aimed at mapping the cellular interactome exploits the enzymatic formation of acylphosphate intermediates on ubiquitin-like small protein domains, namely, prokaryotic ubiquitin-like protein (PUP) (Pearce et al., 2008) and Nedd8 in eukaryotes (Kamitani et al., 1997). PUP and Nedd8, upon activation by specific enzymes, PafA and Ubc12, respectively, form acylphosphates. By fusing proteins capable of forming PUP-acylphosphate or Nedd8-acylphosphate to a POI, proteins that associate with the POI have been identified. Since Nedd8 is an endogenous modification process in cultured mammalian cells, evaluation of the specificity of the tagging process is more complex than PUP. Furthermore, it remains unknown how elevated Nedd8-modification of individual proteins may impinge on native signaling/protein-association networks.

An extension to the Nedd8 approach has also been applied to identification of ligand—protein interactions. In this case, SNAP tag, an epitope tag that reacts irreversibly with a benzyl guanidine (Hill et al., 2016), was fused to an engineered Nedd8-conjugating enzyme Ubc12 that is capable of conjugating a biotinylated Nedd8 to proximal proteins. When a benzyl guanidine tag was fused to a small molecule of interest, such as Dasatinib, a Bcr-Abl/Src kinase inhibitor, known binders of Dasatinib were modified by Nedd8.

The PUP-based method can identify interactions that are very low affinity *in vitro* (maximum *K*_d_ ~ 250 μM). Cell-based studies focused on the interactome of membrane proteins including CD28 (Liu et al., 2018a). Critically, modification by PUP was exclusively at lysine. Furthermore, several new interactions were identified, and these interactors were dependent on the presence of the CD28 C-terminal tail (Figure 3C). The experiments were carried out over a period of 24–36 h, timescale to similar to those used in Bio-ID.

The PUP/Nedd8 methods have strengths and weaknesses similar to Bio-ID as the intermediate formed is an acylphosphate (similar to the acyl-AMP intermediate Di Sabato and Jencks, 1961) and the protein turnover mechanism and kinetics may be similar. However, it is noteworthy that acylphosphate half-lives are variable in solution and biological systems (being dependent likely on enzymatic and metal-catalyzed hydrolysis, to name two variables Di Sabato and Jencks, 1961; Parvez et al., 2018). Thus, careful considerations must be placed on the cellular backgrounds used when comparing the half-lives/diffusion distances of these systems. Although a similar concern applies to phenoxy radicals, such as are generated by APEX, the interaction preferences and modes of interaction/destabilization are different between acyl phosphates and radicals (Parvez et al., 2018). Thus, factors affecting longevity, diffusivity and off-target interactions are likely different between the methods. Interestingly, although both Bio-ID and PafA are ATP-hydrolyzing proteins whose kinetics are readily assessable *in vitro*, these have not been quantitatively compared. It has been shown that PafA is more readily auto PUPylated than BirA is auto-biotinylated (Liu et al., 2018a), but these outputs could be dependent on multiple factors not necessarily intrinsically linked to the activities of the enzymes.

There is also a significant difference in sizes between Nedd8/PUP and biotin. These differences clearly affect several biophysical aspects of the reactive intermediates, including: (1) the diffusion properties of the two molecules (diffusion distances decrease rapidly in cells as a function of size Parvez et al., 2018); (2) how the modified proteins may behave, in terms of association and stability over the long duration required for the experiment; (3) how the cell is affected; (4) the intrinsic reactivity biases of each probe; and yet (5) mean that for Nedd8/PUP both the activating protein AND the substrate's locale can be controlled to zero-in on associations/effects in specific locales.

Finally, it is noteworthy that immediately post synthesis, PafA is able to activate PUP and label interacting proteins. Maturation of T cells (and T-cell receptors, such as CD28) is complex (Wucherpfennig et al., 2010), and it is unknown precisely where upon this maturation process PUP is most readily able to label the CD28 interactome. Almost certainly, PUP is available at the membrane surface where CD28 ultimately resides, but it is unknown if PUP is present at points along the CD28 maturation process. Furthermore, because the dwell time of CD28 is relatively short during its maturation compared to its final localization (Stoops et al., 2015), and PUP-IT is relatively slow to label proteins, it is likely some chaperones are missed even if PUP were to be available at all points along the maturation pathway. APEX, with its faster labeling kinetics and small molecule substrate would likely be able to ID more potential interacting proteins (especially from locales where CD28 does not ultimately reside), especially if used in conjunction with inhibitors. Of course, unlike the small molecule substrate of APEX, PUP can be specifically targeted to specific locales along the CD28 maturation pathway. For instance, attempting to ID CD28 associating proteins using cells expressing ER-localized PUP, would illuminate “only” ER-specific interactors, provided PafA and PUP are functional in the ER.

#### Other Reactive-Molecule-Based Methods and Extensions

Other reactive-molecule-generation methods have recently been disclosed that function similarly to those discussed above, such as reactive N-arylation by N-acyl transferases (Kleinpenning et al., 2018). Although, so far these new techniques have not particularly expanded the repertoire of reactive molecule-based probes, they do have different requirements/cofactors needed for activity. Thus conditions where deployment of these probes is more informative than APEX and BioID may thus not yet have been discovered/assayed. Extensions to APEX and BioID have focused on trying to extract more data from the labeling reactions than simple protein ID using BioID and APEX (Udeshi et al., 2017; Kim et al., 2018). The logic of these extensions goes that a more detailed idea of the interaction region can be gained using such strategies. However, in order for such experiments to work well, the resolution would have to be less than the size of a protein domain 2–5 nm—a scenario unlikely to be easily achievable based on reported diffusion distances (Parvez et al., 2018). There would also have to be assumptions that all residues react equally with these high-energy probes.

## Comparison With Classical Methods to Understand Localization and Association

Protein localization is a critical parameter governing protein function. For instance, many proteins gain new associations, or functions upon translocation leading to important cellular responses. In some cases, the amount of translocation or partitioning of a protein between different organelles can be minimal. For instance, only a 2- to 3-fold *increase* in nuclear RNR-α levels can elicit suppression in DNA synthesis (Fu et al., 2018). Whether such small fold changes could be reliably detected by APEX localization studies and similar methods, in our opinion, remains to be conclusively proven.

The question of where proteins localize has been studied traditionally by immunofluorescence (IF) and fractionation. Both methods are powerful and often give consistent outcomes. These methods are ostensibly quantitative and so in principal can give an idea of relative amounts of protein in one locale over another and can measure even quite small changes.

However, it is worth remembering that traditional methods tend to suffer from limited spatial resolution and low sensitivity. This is for a number of reasons. First, both readouts are typically made by antibodies, so validating specificity through the use of clear controls (knockout/siRNA) are important and in reality in IF and western blotting, background labeling can limit signal to noise. Both methods suffer from intrinsic artifacts: for IF fixing can affect protein localization antigen presentation, whereas use of fluorescent proteins can affect target protein localization; during fractionation proteins can leak from membranes or there can be contamination from unintended structures. Thus, in our opinion at least, perhaps the biggest improvement that reactive labeling methods bring to localization studies is the ability to couple an unambiguous readout (MS) to stringent tagging protocol that is strongly spatially restricted.

There is estimated to be 650,000 protein-protein interactions (PPIs) in human cells, although this number reflects only a fraction of a percent of the total number of possible pairwise interactions (Stumpf et al., 2008). There are likely many more possible associations when one considers protein-DNA/protein-RNA interactions and non-degenerate higher order complexes. Many of these PPIs are robust, with relatively long half-lives and *K*_d_'s in the nanomolar range. Such interactions can be readily assessed by classic methods such as co-IP, native gel, or 2D-PAGE gels. These methods have benefits in that they can be carried out in native cells, tissue, etc. However, requirement for lysing of the cells can introduce artifacts due to dysregulation of cellular compartmentalization, allowing interactions that do not happen in the cell to occur (Fu et al., 2018), or loosing weaker interactions (French et al., 2016). Weaker/more transient associations can be studied by semi-classical methods such as cross-linking (either chemical or UV). Crosslinking methods have the benefit of “trapping” the complex in the cell, prior to lysis, giving more confidence of cellular relevance, and eliminating the possibility of post lysis association. However, the use of reactive cross-linkers also potentially brings in possibilities of off-target cross-linking, can perturb cellular homeostasis, can mask epitopes, and may not be compatible with other transformations/experimental protocols. The reaction products of cross-linking experiments are also complex aggregates that require extensive verification and (typically) excellent antibodies that have been rigorously validated. However, oftentimes protein complexes/aggregates can be resolved using SDS-PAGE, allowing for identification of hetero/homo-dimers and/or higher-order aggregates to be assigned with reasonable accuracy (Aye et al., 2012).

Even though post-lysis associations are minimized by cross linking, there is little information offered concerning where in the cell this association occurs. This can be addressed by imaging experiments. Fluorescence colocalization of FP, or otherwise tagged proteins, or immunofluorescence has been used to visualize associations in live cells (Pedley and Benkovic, 2017), as has FRET (Kenworthy, 2001) and similar methods (Coffey et al., 2016). The use of proximity ligation (Fredriksson et al., 2002; Bellucci et al., 2014), which is read out via immunofluorescence on fixed cells, is also increasing. This method uses DNA-tagged antibodies that when in “close” proximity (40 nm) can template a rolling PCR reaction, allowing for puncta to be observed in specific cellular compartments where an association occurs. This method is signal amplifying, and hence very sensitive. However, since the distance covered (40 nm) by this method is much larger than most proteins, resolution is likely insufficient to “prove” a “direct” interaction.

There are numerous genetic methods to probe PPIs. The most commonly investigated method is the yeast-2-hybrid (Y2H) assay (Vidal and Fields, 2014). This method uses a split transcription factor one terminus of which is fused to a bait protein, and the other terminus of which is typically fused to a series of test proteins. Pairwise combinations of the bait and each test construct are expressed in yeast. When the bait and a test protein interact, the split transcription factor is able to form a viable protein, and typically drives transcription of a gene required for survival, such that only cells expressing proteins that interact with the test protein survive. Aside from the requirement to use ectopic protein and the fact that the native proteins are not used, criticism has been levied at this method because yeast is not a similar environment to human cells in terms of complexity, organelle structure and the posttranslational modifications it is capable of. Interactions must also happen in the nucleus. Furthermore, many Y2H methods are based off a 2 micron-plasmid system (Chan et al., 2013) that gives high expression of each protein, which “may” provide false positives. However, false positives are clearly not as detrimental as false negative, which are also abundant due to incomplete coverage of screening libraries, incomplete expression and poor folding. The use of autosomally replicating sequence-containing plasmids can also alleviate the issue of high protein expression/high copy number (Newlon and Theis, 1993).

Y2H has been extended to mammalian cells, where more complex modifications are possible, but many of the same issues remained, and the library generations are arguably more complex. Non-allelic non-complementation is a screening method that looks for unexpected non-complementation (i.e., where a cross of two strains with mutations to different genes do not give viable offspring) and can be carried out in numerous organisms (Firmenich et al., 1995; Rancourt et al., 1995; Yook et al., 2001). The likely explanation for such an effect is that proteins reside in the same pathway, and commonly these proteins form a complex that is so depleted in the double heterozygote complementation is not possible. Although this is clearly an indirect assay, it has proven very informative and variations of this assay have been used to uncover interesting aspects of cancer biology (Davoli et al., 2013). Aside from these in-cell-relevant experiments, phage display has also been used for HT-protein protein interaction screening (Gibney et al., 2018). This method is of course sensitive and accurate. However, it lacks the ability to be employed in cells (Kokoszka and Kay, 2015).

### Chemotype Specific Sensing and Signaling: REX Technologies

**Figure 4:**
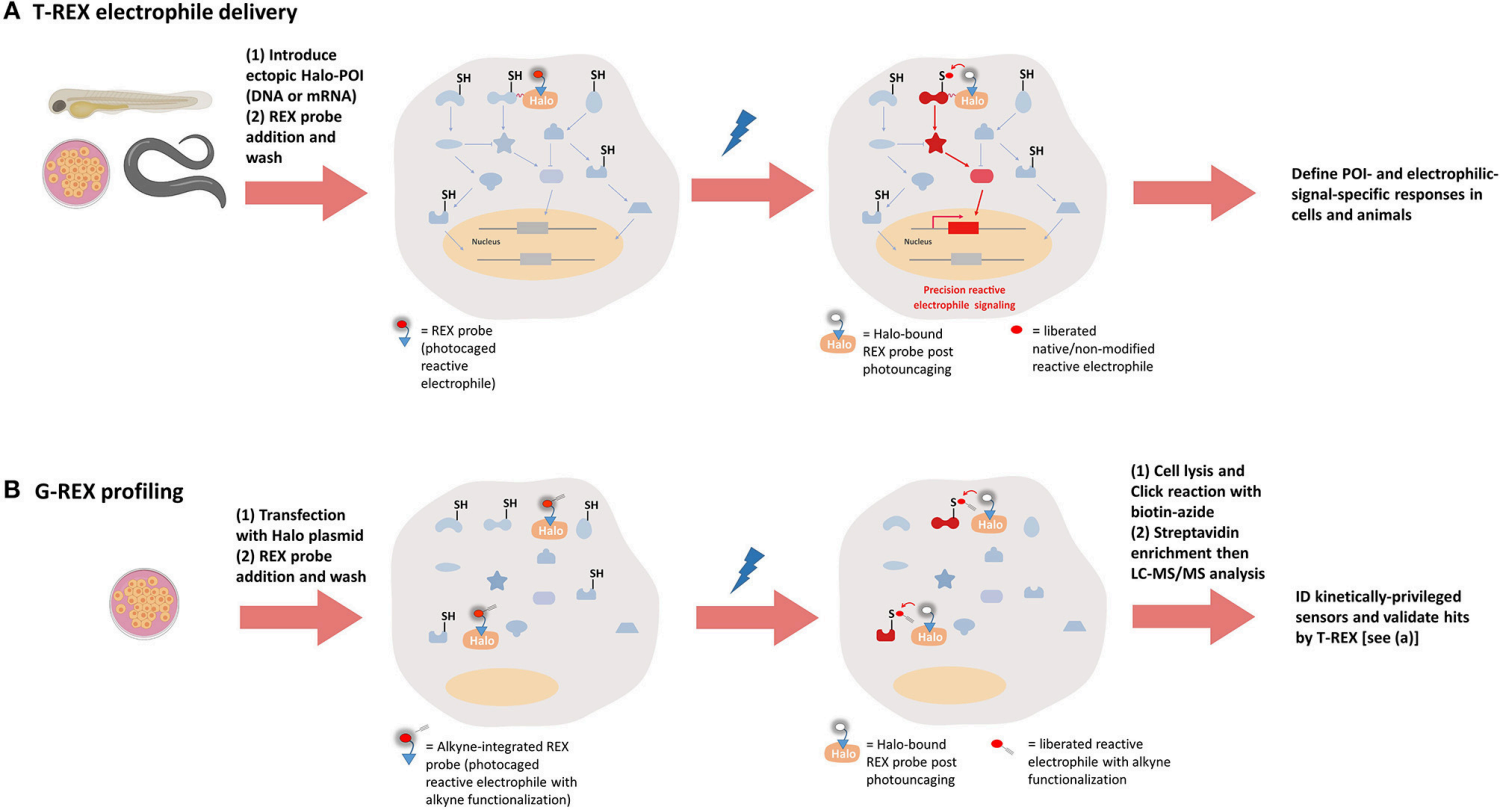
REX technologies to interrogate precision electrophile signaling (T-REX) and mine kinetically-privileged sensors (KPSs) to specific reactive electrophilic species (RES) (G-REX). **(A)** T-REX electrophile delivery. A functional Halo-POI fusion protein is expressed either transiently or stably in live cells, worms, or larval fish. Treatment of these living models with a bio-inert REX probe [photocaged-RES (with or without alkyne functionalization)] (1–25 μM, 1–2.5 h, depending on the system) results in stoichiometric covalent binding of the probe to Halo. After several rounds of exchange with fresh growth media/buffer containing no probe (to washout the unbound REX probe), light exposure (1–3 min, 365 nm, 0.3–5 mW/cm^2^, depending on the system) liberates a specific RES of choice (with or without alkyne-modification) within the microenvironment of Halo-POI, thereby giving the POI the first refusal to the RES. Labeling occurs provided the POI is a KPS of this RES. Provided the resulting substoichiometric RES-modification of the POI is sufficient to elicit either gain-of-function or dominant-loss-of-function signaling responses, T-REX presents a unique means to directly link target-engagement to function. [We define such sensors that can elicit dominant responses at low-occupancy as privileged first responders (PFRs)]. When the alkyne-modified version of the probe is used, the magnitude of measured responses can be quantitatively correlated with the POI-target-occupancy (by fluorescence-gel-based analysis following Click coupling of the alkyne-functionalized-RES-modified-POI with an azido-fluorophore). **(B)** *G-REX profiling*. G-REX enables genome-wide direct ID of KPSs under controlled and RES-limited conditions. Cells ectopically expressing HaloTag-protein are treated with the same REX probe used in T-REX (but the alkyne-modified version) under conditions similar to those deployed in T-REX. Without fusing Halo to any proteins, G-REX approach that allows for user-defined time-, dose-, and locale-controlled release of a specific RES is set to directly capture (localized) native sensors (i.e., KPSs) most responsive to the liberated RES, at low-occupancy covalent RES-modifications. Cell lysis and click coupling with biotin-azide followed by streptavidin enrichment engender RES-bound KPS(s) to be ID'ed by digest LC-MS/MS. The resultant top hits can be functionally validated using T-REX **(A)**.

REX technologies developed by our laboratory were ultimately aimed at studying the signaling function of reactive electrophilic species (RES) in living systems with individual-protein specificity and in precise space and time (Figure 4) (Fang et al., 2013; Lin et al., 2015; Parvez et al., 2015, 2016; Long et al., 2017a,b, 2018a; Hall-Beauvais et al., 2018; Surya et al., 2018; Zhao et al., 2018). The method uses custom-designed bi-functional small-molecule probes [such as Ht-PreHNE for controlled release of a native electrophile 4-hydroxynonenal (HNE)]. One terminus of the probe binds HaloTag irreversibly by virtue of a pendant alkylchloride function. The other end of the bi-functional probe delivers a payload of a specific reactive electrophilic species, e.g., HNE, upon light illumination (*t*_1/2_ of release for various enals/enone-derived electrophiles, <1 min) (Lin et al., 2015). Upon RES liberation, sensor proteins responsive to a given RES have to rapidly intercept the RES prior to diffusion and/or degradation/metabolism (Liu et al., 2019). Thus, the concept underlying REX technologies is unusual in that it harnesses intrinsic “reactivity/affinity-matching” between the released ligand and (a) POI(s) (Long and Aye, 2016, 2017; Long et al., 2016, 2017c; Parvez et al., 2018; Poganik et al., 2018; Liu et al., 2019). HaloTag-targetable photocaged probes such as Ht-PreHNE (1–20 μM) are tolerated by cells for > 2 h, and worms/developing fish for several days (Parvez et al., 2016; Long et al., 2017a,b, 2018a; Hall-Beauvais et al., 2018; Surya et al., 2018; Zhao et al., 2018). Ht-PreHNE does not affect DNA damage response, ubiquitination, and several other essential processes in cells and fish (Parvez et al., 2016; Long et al., 2017a,b; Zhao et al., 2018).

We discuss below two different REX technologies, as well as potential or yet-unnoticed shortcomings of the method.

#### T-REX: Target-Specific Reactive Small-Molecule Sensing and Signaling

T-REX (Figure 4A) uses a HaloTag-POI fusion to give the specific POI first refusal for the RES (e.g., HNE) photouncaged from Halo (Fang et al., 2013; Lin et al., 2015; Parvez et al., 2015, 2016; Long et al., 2017a,b, 2018a; Hall-Beauvais et al., 2018; Surya et al., 2018; Zhao et al., 2018). In this way, a specific POI, providing it is HNE-sensitive, can be HNEylated in the backdrop of a largely unperturbed cell. T-REX gives relatively high RES-occupancy of a specific POI, but incurs very little RES-modification/stress of the total proteome (Parvez et al., 2016; Long et al., 2017a,b; Zhao et al., 2018). Thus, T-REX is also a highly spatially-restricted method and has proven to be compatible with numerous other chemical biology/genetic techniques. Finally, because individual POIs are modified, functional downstream responses elicited as a consequence of specific POI—RES interaction can be read out. Interestingly, proteins that are appreciably modified by HNE under T-REX tend to undergo phenotypically-dominant effects as a consequence of substoichiometric-HNEylation (Lin et al., 2015; Parvez et al., 2015, 2016; Long and Aye, 2017; Long et al., 2017b; Zhao et al., 2018). Thus, T-REX has established that some proteins are wired to react rapidly with HNE and to modulate signaling at fractional occupancy. We have dubbed such proteins privileged first responders (PFRs) (Long and Aye, 2017; Parvez et al., 2018; Poganik et al., 2018; Zhao et al., 2018; Liu et al., 2019). Using T-REX, HNEylation, at individual protein-specific levels, has been shown to impact numerous critical signaling subsystems and pathway intersections, including ubiquitination (Zhao et al., 2018) and phosphorylation (Long et al., 2017b).

The POI-specific nature of T-REX renders the method not particularly high-throughput. G-REX (vide infra) (Zhao et al., 2018) can assume this role if it is needed. Critically, because T-REX uses ectopic expression, RES-labeling and downstream signaling require the HaloTag protein to be fused to POI; and expressing the POI and HaloTag separately and replicating T-REX in this “split” control system ablates both the POI RES-modification and signal propagation downstream (Lin et al., 2015; Parvez et al., 2015, 2016; Long et al., 2017b). Similar controls were recently introduced and shown to be effective for APEX2 (Ariotti et al., 2015, 2018). We have also identified point mutants that are enzymatically or functionally active but do not sense the RES delivered under T-REX conditions (Long et al., 2017b; Surya et al., 2018; Zhao et al., 2018). Notably, these mutants are also refractory to downstream signaling changes induced upon T-REX (Long et al., 2017b; Surya et al., 2018; Zhao et al., 2018).

T-REX has found application to several model organisms, such as *C. elegans* and larval zebrafish (Long et al., 2017b, 2018a; Hall-Beauvais et al., 2018; Zhao et al., 2018). G-REX has as yet not been so applied. T-REX was used in fish embryos to study the effects of HNEylation of two different sensor proteins, Ube2V2 (Poganik et al., 2018; Zhao et al., 2018) and Akt3 (Long and Aye, 2017; Long et al., 2017b). It was noted that in these systems, expression of the transgenes was similar to that of the endogenous proteins (Long et al., 2017b; Zhao et al., 2018), rendering the systems more “natural” than that in cultured cells where the level of Halo-POI-overexpression was significant. Satisfyingly in both cases, delivery and downstream signaling was observed in zebrafish similarly to cell culture. However, because of the implicit requirement of UV-light that is poorly tissue-penetratable, whole organism studies with T-REX on, for instance, mice or adult fish, are not yet possible. This current limitation would not restrict use in certain organs like the brain or blood, however. Two-photon-based photocages would render REX technologies more broadly compatible and would also lower the overall impact of the method on UV-sensitive molecules/processes, such as DNA-synthesis/repair and RNA regulation.

#### G-REX: Genome-Wide Assay for Protein Reactivity With Specific Electrophiles

G-REX (Figure 4B) was established to address limitations underlying with the existing RES-sensor profiling strategies, which rely upon high doses of reactive covalent chemicals for long periods of time. Such flooding strategies tend to incur significant off-target effects due to mass action. These approaches, although they likely achieve high occupancy and modification of multiple potential targets, also affect physiology through, for instance, perturbing cellular redox environment, and inducing stress and apoptosis. RES-permeability, intracellular distribution, metabolism, and specific subcellular redox environments, etc., altogether render the consequences of cell treatment by a reactive molecule such as HNE highly context dependent.

G-REX is designed to release a small, defined pulse of (alkyne-functionalized) RES [e.g., ~5 μM of HNE over 2–5 min in HEK293T cells with ubiquitous Halo expression (Zhao et al., 2018)]. Under these controlled conditions, PFRs to HNE are identified. HNEylated proteins are biotinylated by Click coupling with azido-biotin, precipitation, resolubilization, and streptavidin enrichment followed by mass spectrometry. Using this approach, several PFRs to HNE, including Ube2V2 and Ube2V1, were identified as well as numerous known HNE sensors. Importantly, any enriched hits from G-REX can be validated for HNE-sensing and HNEylation-specific signaling function using T-REX. By contrast, G-REX is not intended to study downstream signaling.

Using G-REX—T-REX coupled strategy, Ube2V2 and Ube2V1 were validated to be HNE-sensitive and modification impact respective signal propagation downstream (Zhao et al., 2018). Several biochemical methods further document these findings. Thus G-REX is an unusual strategy in that it is a global method that aims to achieve only low-occupancy on-target proteins (Liu et al., 2019). Its spatial resolution is currently unknown, although HaloTag itself has been successfully localized to specific subcellular compartments. It remains unknown how diffusive/reactive HNE is, which may intrinsically limit this method's utility to organelle-specific release.

G-REX has several method-specific limitations. First, G-REX only releases a brief and low concentration pulse of RES. Thus, G-REX is a “target-poor” strategy and could potentially miss some privileged sensors. Such issues can be circumvented by repeating experiments numerous (3 or more) times and further integrating quantitative proteomics such as SILAC (Ong et al., 2002) or TMT-labeling (Thompson et al., 2003). However, MS analysis is costly and time consuming and these constraints should be considered when planning/choosing G-REX. To enable target ID, an alkyne-functionalized variant of native RES is used in G-REX. For lipid-derived electrophiles (LDEs), alkyne tagging is minimally (if at all) invasive, although alkynylated versions of many drugs have been successfully deployed for successful target ID (Wright and Sieber, 2016; Parker et al., 2017). Radioisotope tagging or antibody affinity methods present alternatives to alkyne. However, antibodies are much lower sensitivity than alkyne-based Click coupling/enrichment, and radioisotope incorporation may still prove difficult to apply to highly reactive electrophiles, where there is significant background radioactivity, especially given the low occupancy of RES-modification that underlies G-REX. To users' benefit, biotin/streptavidin-based enrichment permits the non-alkynylated electrophile to be used as an “ideal” control for comparison.

## Validations and Current Limitations

REX-probes currently rely on UV-illumination to liberate RES, admittedly for a short period of time and at low-power light sources that the data show does not affect the cell/animal in any appreciable way (Parvez et al., 2016; Long et al., 2017a,b; Zhao et al., 2018). Second, REX-platforms require ectopic expression of HaloTagged-POI (in T-REX) or HaloTag alone (in G-REX) to enable localization and concentration of probe (e.g., Ht-PreHNE) [and liberated RES (e.g., HNE)]. The effects of HaloTag protein on cellular functions are not clearly known, although HaloTag has been applied to numerous systems with little negative effects reported (England et al., 2015).

Notably, identical modification/signaling outcomes are achieved irrespective of N- vs. C-terminal HaloTagging on the POI in T-REX (Lin et al., 2015; Parvez et al., 2016). This outcome indicates that the origin of HNE-liberation is not particularly relevant to sensing. Thus, it can be inferred that T-REX is mimicking genuinely what happens post entry of the liberated RES into the solvent cage of POI fused to Halo. However, we are still unsure whether solvent cage entry is rate-limiting for POI-modification, and if reorganization may cause unanticipated issues that affect efficacy of for instance, POI RES-sensing, or conformational properties of ligand and POI. These concerns have been partially addressed by assaying *in vitro* relative rates of HNEylation of POIs identified to be highly RES-reactive by T-REX in cells/animals (Long et al., 2017b; Surya et al., 2018; Zhao et al., 2018). All sensors assayed were found to be uniquely HNE-sensitive. T-REX assays on POIs identified through G-REX agree with these conclusions (vide infra).

Third, photocaged probes, such as Ht-PreHNE, may be subject to inherent biases (intrinsic concern for any small-molecule probe). Beyond deploying various REX-technical controls and hypomorphic sensing-defective functional mutants (Long et al., 2017b; Zhao et al., 2018), the *in vitro* and other RES bolus dosing experiments in cells discussed above help assuage this worry (Surya et al., 2018). Improved photocaging strategies are presently being undertaken to further limit the possibility of artifacts.

## Non-tethered Approaches

All the above methods share the unifying theme that a “minor” perturbation to typical signaling pathways occurs. Critically, T-REX and G-REX use an ectopic protein anchor to ensure such a system is maintained as much as possible, i.e., the Halo protein serves in part to allow washout of excess probe, ensuring any probe-specific perturbations of the system (due to the lipid fragment of the probe, for instance) can be removed. Similar techniques using non-localized/tethered probes have been applied to mechanistic analysis and target ID, using dual photocaging (Höglinger et al., 2017). However, in these methods, the photocaged cannot be completely washed away, due to having no probe-anchoring device, such as Halo. Organelle specificity has been instead achieved by chemical means, such as fusing triphenylphosphine to direct the probe to the mitochondria (Wagner et al., 2018), and many pioneering contributions have been made in this arena. The probe concentrations and those of the released lipids are difficult to normalize under these systems, and are likely not readily tunable or comparable between different cells. When using an ectopic protein, as the ectopic protein expression can be calculated, and the amount of precursor on the protein can also be assessed, these values are much more readily normalized. To some extent, the adverse effects of the excess probe and the uncaged species can be circumvented by irradiating specific sections of a cell. However, this approach is restricted to single or a few cell-based analyses.

## Outlook

Our aim in this review is 2-fold: to stimulate discussion on the fundamentals of chemical biology methods; and to highlight methods development at the boundary of chemistry and biology with the focus on emerging chemistry-driven perturbation methods that shed light on the biological locale/interactome and signaling consequences. It is at this intersection of biochemical/enzymological and organic chemistry disciplines where we feel chemical biology is most useful and where as a field we need to go. Improvement and further expansion should be built on our better understanding and appreciation of where the field currently is in terms of limitations that it faces and successes it has had, on our conscious and responsible use of methods and understanding of systems to apply them, and on having a firm idea of where the field is going. We strongly believe that chemical biology has the ability to deeply probe complex biological questions but our progress is hampered by reliance on unrealistic models and analogy to former biochemical studies. Using the most relevant model systems will be an enabling step forward in successfully tackling the important problems unsolvable by traditional genetics and biochemistry.

## Author Contributions

MJCL and XL contributed equally to this work. XL contributed to the creation of all figures and tables as well as manuscript editing and formatting. MJCL drafted the manuscript. YA oversaw the manuscript outline, overall direction and planning. All authors contributed to reference collection, selection and final proof.

### Conflict of Interest Statement

G-REX technology had been filed for patent application by Cornell University (USA).

## Footnotes

^1^https://www.nobelprize.org/prizes/medicine/1957/summary/
